# Supplementary material for: Heat adaptation of phage T7 under an extended genetic code
Source: Virus Evol. 2021 Dec 1;7(2):veab100. doi: 10.1093/ve/veab100 (PMC8923235; doi:10.1093/ve/veab100)
Supplement: veab100_Supp [file veab100_supp.zip › Sequencing_Analysis.zip!/Sequencing_Analysis/A3/evidence/MC_SIDE_2_51.html]

BRESEQ :: Evidence


| Predicted mutation | | | | | | |
| --- | --- | --- | --- | --- | --- | --- |
| evidence | seq id | position | mutation | annotation | gene | description |
| MC JC | NC\_001604.1 | 13,278 | Δ1,019 bp | intergenic (–/–) | – / – | –/– |

| Missing coverage evidence... | | | | | | | | | | |
| --- | --- | --- | --- | --- | --- | --- | --- | --- | --- | --- |
|  |  |  | seq id | start | end | size | ←reads | reads→ | gene | description |
| \* | \* | ÷ | NC\_001604.1 | 13278 | 14296 | 1019 | 1612 [523] | [14] 1100 | *–/–* | –/– |

| New junction evidence | | | | | | | | | | | |
| --- | --- | --- | --- | --- | --- | --- | --- | --- | --- | --- | --- |
|  | | seq id | position | reads (cov) | reads (cov) | score | skew | freq | annotation | gene | product |
| \* | ? | NC\_001604.1 | = 13277 | 522 (0.300) | 1073 (0.630) | 291/586 | 0.3 | 80.4% | intergenic (–/–) | *–/–* | –/– |
|  | ? | NC\_001604.1 | 14297 = | 14 (0.010) |  |  |  |  | intergenic (–/–) | *–/–* | –/– |

Only 100 of 1100 total aligned reads displayed.  

`ATGAAGCTAAGACTCGCTCCGCTGTTCACATCTTGAAGAACTTAGGCTGGACTTGGACAAGACAGACTGGCTGGAAGAAACCAGAAGTTACCAAGCTGAGTCATAAGGTGTTCGATAAGGACACTATGACCCACATCAAGGCTGGTGATTGGGTTAAGGTTGACATGGGAGTTGTTGGTGGATACGGCTACGTCCGCTCAGTTAGTGGCAAATATGCACAAGTGTCATACATCACAGGTGTTACTCCACGCGGTGCAATCGTTGCCGATAAGACCAACATGATTCACACAGGTTTCTTGACAGTTGTTTCATATGAAGAGATTGTTAAGTCACGATAATCAATAGGAGAAATCAATATGATCGTTTCTGACATCGAAGCTAACGCCCTCTTAGAGAGCGTCACTAAGTTCCACTGCGGGGTTATCTACGACTACTCCACCGCTGAGTACGTAAGCTACCGTCCGAGTGACTTCGGTGCGTATCTGGATGCGCTGGAAGCCGAGGTTGCACGAGGCGGTCTTATTGTGTTCCACAACGGTCACAAGTATGACGTTCCTGCATTGACCAAACTGGCAAAGTTGCAATTGAACCGAGAGTTCC  >  NC_001604.1/13997‑14596`  

                                                                                                                                                                                                                                                                                                            `|`                                                                                                                                                                                                                                                                                                             
`ggtacatgacaggagtaaggtcgccccttttcgattactgacctactgggttctggcgcactacgccaactatctgatactattagtgcccttgagcgtaatcagcaaggcgataggcctaaccttgtcctcgttcgtattctcaagtgccgctttactggtgatactggtatcgctggctacatggaatacaacaaggaaactggatggcttgaaccatcaagttactcaggggaagaagagtcacactcagagtcaacagactggtccaacgacactgacttctgacaggattcttgac                                                                                                                                                                                                                                                                                                             <  1:51119‑M2/1‑1 (MQ=255)`  
`aggtaaagcacatgaggaaggtcgccccgtttctattactgacctacgtggttctggcgcactacgccaactatctgatactattattgcccttgagcgtaatcagcaaggcgatatgcctaaccttgtcctcgttcgtattctcaagtgccgctttactggtgatactggtatcgctggctacatggaatacaacaaggaaaccggatggcttgaaccatcaagtactcaggggaagaagagtcacactcagagtcaacagactggtccaacgacactgacttctgacaggattcttgac                                                                                                                                                                                                                                                                                                             <  1:149375‑M2/1‑1 (MQ=255)`  
   `aaagcacatgaggaaggtcgccccgtttctagtactgacctacgtggttctggcgcactacgccaactatctgatactattattgccctggagcgtaatcagcaaggcgatatgcctaaccttgtcctcgttcgtattctcaagtgccgctttactggtgatactggtatcgctggctacatggaatacaacaaggaaaccggatggcttgaaccatcaagttactcaggggaagaagagtcacactcagagtcaacagactggtccaacgacactgacttctgacaggattcttgaCAgt                                                                                                                                                                                                                                                                                                          <  1:65415‑M2/4‑1 (MQ=255)`  
           `tgaggaaggtcgccccgtttctattactgacctacgtggttctggcgcactacgccaactatctgatactattattgcccttgagcgtaatccgcaaggcgatatgcctaaccttgtcctcgttcgtattctcaagtgccgctttactggtgatactggtatcgctggctacatggaatacaacaaggaaaccggatggcttgaaccatccagttactcaggggaagaagagtctcactcagagtcaacagactggtccaacgacactgacttcttacaggtttcttgaCAGTTGTTTCat                                                                                                                                                                                                                                                                                                  >  1:32711‑M2/290‑301 (MQ=255)`  
           `tgaggaaggtcgccccgtttctattactgacctacgtggttctggcgcactacgccaactatctgatactattattgcccttgagcgtaatcagcaaggcgatatgcctaaccttgtcctcgttcgtattctcaagtgccgctttactggtgatactggtatcgctggctacatggaatacaacaaggaaaccggatggcttgaaccatcaagttactcaggggaagaagagtcacactcagagtcaacagactggtccaacgacactgacttctgacaggattcttgaCAGTTGTTTCat                                                                                                                                                                                                                                                                                                  >  1:97248‑M2/290‑301 (MQ=255)`  
            `gaggaatgtcgccccgtttctattactgacctacgtggttctggcgcactacgccaactatctgatactattattgcccttgagcgtaatcagcaaggcgatatgcctaaccttgtcctcgttcgtattctcaagtgccgctttactggtgatactggtatcgctggctacatggaatacaacaaggaaaccggatggcttgaaccatcaagttactcaggggaagaagagtcacactcagagtcaacagactggtccaacgacactgccttctgacaggattcttgaCAGTTGTTTcatt                                                                                                                                                                                                                                                                                                 >  1:203142‑M2/289‑300 (MQ=255)`  
            `gaggaaggtcgccccgtttctattactgacctacgtggttctggcgcactacgccaactatctgatactattattgcccttgagcgtaatcagcaaggcgatatgcctaaccttgtcctcgttcgtattctcaagtgccgctttactggtgatactggtatcgctggctacatggaatacaacaaggaaaccggatggcttgaaccatcaagttactcaggggaagaagagtcacactcagagtcaacagactggtccaacgacactgacttctgacaggattcttgaCAGTTGTTTCata                                                                                                                                                                                                                                                                                                 >  1:52255‑M2/289‑301 (MQ=255)`  
                 `aggtcgccccgtttctattactgacctacgtggttctggcgcactacgccaactatctgatactattattgcccttgagcgtaatcagcaaggcgatatgcctaaccttgtcctcgttcgtattctcaagtgccgctttactggtgatactggtatcgctggctacatggaatacaacaaggaaaccggatggcttgaaccatcaagttactcaggggaagaagagtcacactcagagtcaacagactggtccaacgacactgacttctgacaggattcttgaCAGTTGTTTCATATGAag                                                                                                                                                                                                                                                                                            <  1:94657‑M2/18‑1 (MQ=255)`  
                             `ttcttttactgacctacgtggttctggcgcactacgccaactatctgatactattattgcccttgagcgtaatcagcaaggcgatatgcctaaccttgtcctcgttcgtattctcaagtgccgctttactggtgatactggtatcgctggctacatggaatacaacaaggaaaccggatggcttgaaccatcaagttactcaggggcagaagagtcacactcagagtcaacagactggtccaacgacactgacttctgacaggattcttgaCAGTTGTTTCATATGAAGAGATTGTTAAGt                                                                                                                                                                                                                                                                                >  1:87288‑M2/272‑301 (MQ=255)`  
                             `ttctattactgacctacgtggttctggcgcactacgccaactatctgatactattattgcccttgagcgtaatcagcaaggcgatatgcctaaccttgtcctcgttcgtattctcaagtgccgctttactggtgatactggtatcgctggctacatggaatacaacaaggaaaccggatggcttgaaccatcaagttactcaggggaagaagagtcacactcagagtcaacagactggtccaacgacactgacttctgacaggcttcttgaCAGTTGTTTCATATGAAGAGATTGTTAAGt                                                                                                                                                                                                                                                                                >  1:84901‑M2/272‑301 (MQ=255)`  
                              `tctattactgacctacgtggttctggcgcactacgccaactatctgatactattatttccctttatcgtaatcagcaaggcgatatgcctaaccttttcctcgttcgtattctcaagtgccgctttactggttatactggtatcgctggctacatggaatacaacaaggacaccggatggcttgaaccatcaagttactcaggggaagaagagtcacactcagagtcaacagactggtccaacgacactgacttctgacaggattcttgaCAGTTGTTTCATATGAATAGATTCTTAAGTc                                                                                                                                                                                                                                                                               >  1:46593‑M2/271‑301 (MQ=255)`  
                                  `ttactgacctacgtggttcttgcgcactacgccaactatctgatactattattgcccttgagcgtaatcagcaaggcgatatgcctaaccttgtcctcgttcgtattctcaagtgccgctttactggtgatactggtatcgctggctacatggaatacaacaaggaaaccggatggcttgaaccatcaagttactcaggggaagaagagtcacactcagagtcaacagactggtccaacgacactgacttctgacaggattcttgaCAGTTGTTTCATATGAAGATATTGTTAAGTCACGa                                                                                                                                                                                                                                                                           >  1:225047‑M2/267‑301 (MQ=255)`  
                                  `ttactgacctacgtggttctggcgcactacgccaactatctgatactattattgccctttagcgtaatcagcaaggcgatatgcctaaccttgtcctcgttcgtattctcaagtgccgctttactggtgatactggtatcgctggctacatggaatacaacaaggaaaccggatggcttgaaccatcaagttactcaggggaagaagagtcacactcagcgtcaacagactggtccaacgacactgacttctgacaggattcttgaCAGTTGTTTCATATGAAGAGATTGTTAAGTCACGa                                                                                                                                                                                                                                                                           >  1:310416‑M2/267‑301 (MQ=255)`  
                                       `ggagatgtgtataagagacagctacgccaactatctgatactattattgcccttgggcgtaatcagcaaggcgatatgcctaaccttgtcctcgttcgtattctaaagtgccgctttactggtgatactggtatcgctagctacatggaatacaacaaggaaaccggatggcttgaaccatcaagttactcaggggaagaagagtcacactcagagtcaacagactggtccaacgacactgacttctgacaggattcttgaCAGTTGTTTCATATGAAGAGATTGTTAAGTCACGATAATc                                                                                                                                                                                                                                                                      <  1:330738‑M2/40‑1 (MQ=255)`  
                                       `gacctacgtggttctgacgcactacgccaactatctgatactattattgcccttgagcgtaatcagcaaggcgatatgcctaaccttgtcctcgttcgtattctcaagtgccgctttactggtgatactggtatcgctggctacatggaatacaacaaggaaaccggatggcttgaaccatcaagttactcaggggaagaagagtcacactcagagtcaacagactggtccaacgacactgacttctgacaggattcttgaCAGTTGTTTCATATGAAGAGATTGTTAAGTCACGATAATc                                                                                                                                                                                                                                                                      >  1:261921‑M2/262‑301 (MQ=255)`  
                                            `tgtgtataagagacagctacgccaactatctgatactattattgcccttgagcgtaatcagcaaggtgatatgcctaaccttgtcctcgttcgtattctcaagtgccgctttactggtgatactggtatcgctggctacatggaatacaacaaggaaaccggatggcttgaaccatcaagttactcaggggaagaagagtcacactcagagtcaacagactggtccaacgacactgacttctgacaggtttcttgaCAGTTGTTTCATATGAAGAGATTGTTAAGTCACGATAATCAATAg                                                                                                                                                                                                                                                                 <  1:33823‑M2/45‑1 (MQ=255)`  
                                               `agttgcttgcgcactacgccaactatctgatactattattgcccttgagcgtaatcagcaaggcgatatgcctaaccttgtcctcgttcgtattctcaagtgccgctttactggtgatactggtatcgctggctacatggaatacaacaaggaaaccggatggcttgaaccatcaagttactcaggggaagaagagtcacactcagagtcaacagacttgtccaacgacactgacttctgacaggattcttgaCAGTTGTTTCATATGAAGAGATTGTTAAGTCACGATAATCAATAGgag                                                                                                                                                                                                                                                              <  1:235404‑M2/48‑1 (MQ=255)`  
                                                 `gttcttgcgcactacgccaactatctgatactattattgcccttgagcgtaatcagcaaggcgatatgcctaaccttgtcctcgttcgtattctcaagtgccgctttactggtgatactggtatcgctggctacatggaatacaacaaggaaacgggatggcttgaaccatcaagttactcaggggaagaagagtcacactcagagtcaacagactggtccaacgacactgacttctgacaggcttcttgaCAGTTGTTTCATATGAAGAGATTGTTAAGTCACGATAATCAATAGGAGaa                                                                                                                                                                                                                                                            >  1:24589‑M2/252‑301 (MQ=255)`  
                                                    `ctggcgcactacgccaactatctgatactattattgcccttgagcgtaatcagcaaggcgatatgcctaaccttgtcctcgttcgtattctcaagtgccgctttactggtgatactggtatcgctggctacatggaatacaacaaggaaaccggatggcttgaaccatcaagttactcaggggaagaagagtcacactcagagtcaacagactggtccaacgacactgacttctgacaggattcttgaCAGTTGTTTCATATGAAGAGATTGTTaagtcacgataatcctgtctcttatac                                                                                                                                                                                                                                                         >  1:147257‑M2/249‑288 (MQ=255)`  
                                                         `tgtataagagacagatctgatactattattgcccttgagcgtaatcagcaaggcgatatgcctaaccttgtcctcgttcgtattctcaagtgccgctttactggtgatactggtatcgctggctacatggaatacaacaaggaaaccggatggcttgaaccatcaagttactcaggggaagaagagtcacactcagagtcaacagactggtccaacgacactgacttctgacaggattcttgaCAGTTGTTTCATATGAAGAGATTGTTAAGTCACGATAATCAATAGGAGAAATAAatat                                                                                                                                                                                                                                                    <  1:269403‑M2/58‑1 (MQ=255)`  
                                                         `ccactacgccaactatctgataatattattgcccttgagagtaatcagcaaggcgatatgcctaaccttgtcctcgttcgtattctcaagtgccgctttactggtgatactggtatcgctggctacatggaatacaacaaggaaaccggatggcttgaaccattaagttactcaggggaagaagagtcacactcagagtcaacagactggtccaacgacactgacttctgacaggattcttgaCAGTTGTTTCATATGAAGAGATTGTTAAGTCACGATAATCAATAGGAGAAATCAatat                                                                                                                                                                                                                                                    <  1:145224‑M2/58‑1 (MQ=255)`  
                                                          `cactacgccaactatctgatactattattgcccttgagcgtaatcagcaaggcgatatgcctaaccttgtcctcgttcgtattctcaagtgccgctttactggtgatactggtatcgctggctacatggaatacaacaaggaaaccggatggcttgaaccatcaagttactcaggggaagaagagtcacactcagagtcaacagactggtccaacgacactgacttctgacaggattcttgaCAGTTGTTTCATATGAAGAGATTGTTAAGTCACGATAATAAATAGGAGAAATACATATg                                                                                                                                                                                                                                                   >  1:48665‑M2/243‑301 (MQ=255)`  
                                                            `ctacgccaactatctgatactattattgcccttgagcgtaatcagcaaggcgatatgcctaaccttgtcctcgttcgtattctcaagtgccgctttactggtgatactggtatcgctggctacatggaatacaacaaggaaaccggatggcttgaaccatcaagttactcaggggaagaagagtcacactcagagtcaacagactggtccaacgacactgacttctgacaggattcttgaCAGTTGTTTCATATGAAGAGATTGTTAAGTCACGATAATCAATAGGAGAAatcaatagtgt                                                                                                                                                                                                                                                 >  1:118036‑M2/241‑297 (MQ=255)`  
                                                            `ctacgccaactatctgatactattattgccattgagcgtaatcagcaaggcgatatgcctaaccttgtcctcgttcgtattctcaagtgccgctttactggtgatactggtatcgctggctacatggaatacaacaaggaaaccggatggcttgaaccatcaagttactcaggggaagaagagtcacactcagagtcaacagactggtccaacgacactgacttctgacaggattcttgaCAGTTGTTTCATATGAAGAGATTGTTAAGTCACGATAATCAATAGGAGAAATCAATATGAt                                                                                                                                                                                                                                                 <  1:163126‑M2/61‑1 (MQ=255)`  
                                                                    `gctatctgatactattattgcccttgagcgtaatcagcaaggcgatatgcctaaccttgtcctcgttcgtattctaaaatgccactttactggggatactggtatcgctggctacatggaatacaacaaggaaaccggatggcttgaaccatcaagttactcaggggaagaagagtcacactaagagtcaacagactggtccaacgacactgacttctgacaggattcttgaCAGTTGTTTCATATGAAGAGATTGTTAAGTCACGATAATCAATAGGAGAAATCAATATGATCGTTTCTg                                                                                                                                                                                                                                         <  1:292524‑M2/69‑1 (MQ=255)`  
                                                                     `ctatctgatactattattgcccttgagcgtaatcagcaaggcgatatgcctaaccttgtcctcgttcgtattctcaagtgccgctttactggtgatactggtatcgcttgctacatggaatacaacaaggaaacaggatggcttgaaccatcaagttactcaggggaagaagagtcacactcagagtcaacagactggtccaacgacactgacttctgacaggattcttgaCAGTTGTTTcatatgaagagattgttaagtcacgataatcctgtctcttatacacatctccgagcccacg                                                                                                                                                                                                                                        >  1:298410‑M2/232‑271 (MQ=255)`  
                                                                       `atctgatactattattgcccttgagcgtaatcagcaaggcgatatgcctaaccttgtcctcgttcgtattctcaagtgccgctttactggtgatactggtatcgctggctacatggaatacaacaaggaaaccggatggcttgaaccatcaagttactcagtggaagaagagtcacactcagagtcaacagactggtccaacgacactgacttctgacaggattcttgaCAGTTGTTTCATATGAAGAGATTGTTAAGTCACGATAATCAATAGGAGCAATCAATATAATCGTTTCTGACa                                                                                                                                                                                                                                      >  1:271335‑M2/230‑301 (MQ=255)`  
                                                                       `atctgatactattattgcccttgagcgtaatcagcaaggcgatatgcctaaccttgtcctcgttcgtattctcaagtgccgctttactggtgatactggtatcgctggctacatggaatacaacaaggaaaccggatggcttgaaccatcaagttactcaggggaagaagagtcacactcagagtcaacagactggtccaacgacactgacttctgacaggattcttgaCAGTTGTTTCATATGAAGAGATTGTTAAGTCACGATAATCAATAGGAGAAATCAATATTATCGTTTCTGACa                                                                                                                                                                                                                                      >  1:209901‑M2/230‑301 (MQ=255)`  
                                                                       `atctgatactattattgcccttgagcgtaatcagcaaggcgatatgcctaaccttgtcctcgttcgtattctcaagtgccgctttactgatgatactggtatcgctggctacatggaatacaacaaggaaaccggatggattgaaccatcaagttactcaggggaagaagagtcacactcagagtcaacagactggtccaacgacactgacttctgacaggattcttgaCAGTTGTTTCATATGAAGAGATTGTTAAGTCACGATAATCAATAGGAGAAATCAATATGATCGTTTCTGACa                                                                                                                                                                                                                                      >  1:68400‑M2/230‑301 (MQ=255)`  
                                                                       `atctgatactattattgccattgagcgtaatcagcaaggcgatatgcctaaccttgtcctcgttcgtattctcaagtgccgctttactggtgatactggtatcgctggcaacatggaatacaacaaggaaaccggatggcttgaaccatcaagttactcaggggaagaagagtcacactcagagtcaacagactggtccaacgacactgacttctgacaggattcttgaCAGTTGTTTCATATGAAGAGATTGTTAAGTCACGAAAATCAATAGGAGAAATCAATATGATCGTTTCTGACa                                                                                                                                                                                                                                      <  1:227812‑M2/72‑1 (MQ=255)`  
                                                                             `tactattattgcccttgagcgtaatcagcaaggcgatatgcctaaccttgtcctcgttcgtattctcaagtgccgctttactggtgatactggtatcgctggctacatggaatacaacaaggaaaccggatggcttgaaccatcaagttactcaggggaagaagagtcacactcagagtcaacagactggtccaacgacactgacttctgacaggattcttgaCAGTTGTTTCATATGAAGAGATTGTTAAGTCACGATAATCAATAGGAGAAATCAATATGATCGTTTCTGACATCGAAg                                                                                                                                                                                                                                >  1:58077‑M2/224‑301 (MQ=255)`  
                                                                               `ctattattgcccttgagcgtaatcagcaaggcgatatgcctaaccttgtcctcgttcgtattctcaagtgccgctttactggtgatactggtatcgctggctacatggaatacaacaaggaaaccggatggcttgaaccatcaagttactcaggggaagaagagtcacactcagagtcaacagactggtccaacgacactgacttctgacaggcttcttgaCAGTTGTTTCATATGAAGAGATTGTTAAGTCACGATAATCAATAGGAGAAATCAATCTGATCGTTTCTGACATCGAATCt                                                                                                                                                                                                                              >  1:322700‑M2/222‑301 (MQ=255)`  
                                                                               `ctattattgcccttgagcgtaatcagcaaggcgatatgcctaaccttgtcctcgttcgtattctcaagtgccgctttactggtgatactggtatcgctggctacatggaatacaacaaggaaaccggatggcatgaaccatcaagttactcaggtgaagaagagtcacactcagagtcaacagactggtccaacgacactgacttctgacaggattcttgaCAGTTGTTTCATATGAAGAGATTGTTAAGTCACGATAATCAATAGGAGAAATCAATATGATCGTTTCTGACATCGAAGCt                                                                                                                                                                                                                              >  1:219817‑M2/222‑301 (MQ=255)`  
                                                                                     `ttgcccttgagcgtaatcagcaaggcgatatgcctaaccttgtcctcgttcgtattctcaagtgccgctttactggtgatactggtatcgctggctacacggaatacaacaaggaaaccggatggcttgaaccatcaagttactcaggggaagaagagtcacactcagagtcaacagactggtccaacgacactgacttctgacaggattcttgaCAGTTGTTTCATATGAAGAGATTGTTAAGTCACGATAATCAATAGGAGAAATCAATATGATCGTTTCTGACATCGAAGCTAACGcc                                                                                                                                                                                                                        >  1:80238‑M2/216‑301 (MQ=255)`  
                                                                                       `gcccttgagcgtaatcagcaaggcgatatgcctaaccttgtcctcgttcgtattctcaagtgccgctttactggtgatactggtatcgctggctacatggaatacaacaaggaaaccggatggcttgaaccatcaagttactcaggtgaagaagagtcacactcagagtcaacagactggtccaacgacactgacttctgacaggattcttgaCAGTTGTTTCATATGAAGAGATTGTTAAGTCACGATAatcaataggagaaatcaatatgatcgctgtctcttatacacctctccgagc                                                                                                                                                                                                                      >  1:135379‑M2/214‑276 (MQ=255)`  
                                                                                            `tgagcgtaatcagcaaggcgatatgcctaaccttgtcctcgttcgtattctcaagtgccgctttactggtgatactggtatcgctggctacatggaatacaacaaggaaaccggatggcttgaaccatcaagttactcaggggaagaagagtcacactcagagtcaacagactggtccaacgacactgacttctgacaggattcttgaCAGTTGTTTCATATGAAGAGATTGTTAAGTCACGATAATCAATAGGAGAAATCAATATGATCGTTTCTGACATCGAAGCTAACGCCCTCTTag                                                                                                                                                                                                                 <  1:71831‑M2/93‑1 (MQ=255)`  
                                                                                             `gagcgtaatcagcaaggcgatatgcctaaccttgtcctcgttcgtattctcaagtgccgctttactggtgatactggtatcgctggctacatggaatacaacaaggaaaccggatggcttgatccatcaagttactcaggggaagaagagtcacactcagagtcaacagactggtccaacgacactgacttctgacaggattcttgaCAGTTGTTTCATATGAAGAGATTGTTAAGTCACGATAATCAATAAGAGAAATCAATATGATCGTTTCTGACATCGAAGCTAACGCCCTCTTaga                                                                                                                                                                                                                >  1:100119‑M2/208‑301 (MQ=255)`  
                                                                                              `agcgtaatcagcaatgcgatatgcctaaccttgtcctcgttcgtattctcaagtgccgctttactggtgatactggtatcgctggctacatggaatacaacaaggaaaccggatggcttgaaccatcaagttactcaggggaagaagagtcacactcagagtcaacagactggtccaacgacactgacttctgacaggattcttgaCAGTTGTTTCATATGAAGAGATTGTTAAGTCACGATAATCAATAGGAGAAATCAATATGATCGTTTCTGACATCGAAGCCAACGCCCTCTTagag                                                                                                                                                                                                               <  1:195306‑M2/95‑1 (MQ=255)`  
                                                                                              `agcgtaatcagcaaggcgatatgcctaaccttgtcctcgttcgtattctcaagtgccgctttactggtgatactggtatcgcttgctacatggaatacaacaaggaaaccggatggctttaaccatcaagttactcaggggaagaagagtcacactcagagtcaacagactggtccaacgacactgacttctgacaggattcttgaCAGTTGTTTCATATGAAGAGATTGTTAAGTCACGATAATCAATAGGAGAAATCAATATGATCGTTTCTGACATCGAAGCTAACGCCCTCTTagag                                                                                                                                                                                                               <  1:105511‑M2/95‑1 (MQ=255)`  
                                                                                              `agcgtaatcagcaaggcgatatgcctaaccttgtcctcgttcgtattctcaagtgccgctttactggtgatactggtatcgctggctacatggaatacaacaaggaaaccggatggcttgaaccatcaagttactcaggggaagaagagtcacactcagagtcaacagactggtccaacgacactaacttctgacaggattcttgaCAGTTGTTTCATATGAAGAGATTGTTAAGTCACGATAATCAATAGGAGAAATCAATATGATCGTTTCTGACATCGAAGCTAACGCCCTCTTagag                                                                                                                                                                                                               <  1:169017‑M2/95‑1 (MQ=255)`  
                                                                                              `agagacagcagcaaggcgatatgcctaaccttgtcctcgttcgtattctcaagtgccgctttactggtgatactggtatcgctggctacatggaatacaacaaggaaaccggatggcttgaaccatcaagttactcaggggaagaagagtcacactcagagtcaacagactggtccaacgacactgacttctgacaggattcttgaCAGTTGTTTCATATGAAGAGATTGTTAAGTCACGATAATCAATAGGAGAAATCAATATGATCGTTTCTGACATCGAAGCTAACGCCCTCTTagag                                                                                                                                                                                                               <  1:55726‑M2/95‑1 (MQ=255)`  
                                                                                              `agagaaagcagcaaggcgatatgcctaaccttgtcctcgttcgtattctcaagtgccgctttacgggtgatactggtatcgctggctacatggaatacaacaaggaaaccggatggcttgaaccatcaagttactcaggggaagaagagtcacactcagagtcaacagactggtacaacgacactgacgtctgacaggattcttgaCAGTTGTTTCAGATGAAGAGATTGTTAAGTCACGATAATCAATAGGAGAAATCAATATGATCGTTTCTGACATCGAAGCTAACGCCCTCTTagag                                                                                                                                                                                                               <  1:36805‑M2/95‑1 (MQ=255)`  
                                                                                                `cgtaatcagcaaggcgatatgcctaaccttgtcctcgttcgtattctcaagtgccgctttactggtgatactggtatcgctgactacatggaatacaacaaggaaaccggatggcttgaaccatcaagttactcaggggaagaagagtcacactcagagtcaacagactggtccaacgacactgacttctgacaggattcttgaCAGTTGTTTCATATGAAGAGATTGTTAAGTCACGATAATCAATAGGAGAAATCAATATGATCGTTTCTGACATCGAAGCTAACGCCCTCTTagagag                                                                                                                                                                                                             <  1:286454‑M2/97‑1 (MQ=255)`  
                                                                                                        `acaaggcgatatgcctaatcttgtactcgttcgtattctcaagtgccgctttactggtgatactggtatcgctggctacatggaatacaacaaggaaaccggatggcttgaaccatcaagttactcaggggaagaagagtcacactcagagtcaacagactggcccaacgacactgacttctgacaggattcttgaCATTTGTTTCATATGAAGAGATTGTTAAGTCACGATAATCGATAGGAGAAATCAATATGATCGTTTCTGACATCGAAGCTAACGCCCTCTTAGAGAGCGTCACTa                                                                                                                                                                                                     <  1:138214‑M2/105‑1 (MQ=255)`  
                                                                                                              `ggataagcctaaccttgtccacgttcgtattctcaagtgccgatttactggggatactggtatcgctggctacatggaatacaacaaggaaaccggatggcttgaaccatcaagttactcaggggaagaagagtcacactcagagtcaacagactggtccaacgacactgacttctgacaggattcttgaCAGTTGTTTCATATGAAGAGATTGTTAAGTCACGATAATAAATAGGAGAAATCAATATGATCGTTTCTGACATCAAAGCTAACGCCCTCTTAGAGAGCGTCACTAAGTTcc                                                                                                                                                                                               <  1:10265‑M2/111‑1 (MQ=255)`  
                                                                                                                    `gcctaaccttgtcctcgttcgtattctcaagtgccgctttactggtgatacttgtatcgctggctacatggaatacaacaaggaaaccggatggcttgaaccatcaagttactcaggggaagaagagtcacactcagagtcaacagactggtccaacgacactgacttctgacaggtttcttgaCAGTTGTTTCATATGAAGAGATTGTTAAGTCACGATAATCAATAGGAGAAATCAATATGATCGTTTCTGACATCGAAGCTAACGCCCTCTTAGATAGCGTCACTAAGTTCCACTGCg                                                                                                                                                                                         >  1:190415‑M2/185‑301 (MQ=255)`  
                                                                                                                    `gcctaaccttgtcctcgttcgtattctcaagtgccgctttactggtgatactggtatcgctggctacatggaatacaacaaggaaaccggatggcttgaaccatcaagttactcaggggaagaagagtcacactcagagtcaacagactggtccaacgacactgacttctgacaggattcttgaCAGTTGTTTCATATGAAGAGATTGTTAAGTCACGATAATCAATAGGAGAATTCAATAtgatcgtttctgacatcgaagctaacgccctgtctcttatacacatctccgagaccacga                                                                                                                                                                                         >  1:73415‑M2/185‑272 (MQ=255)`  
                                                                                                                        `aaccgtgtcctcgttcgtattctcaagtgccgctttactggtgatactggtatcgctggctacatggaatacaacaaggaaaccggatggcttgaaccatcaagttactcaggggaagaagagtcacactcagagtcaacagactggtccaacgacactgacttctgacaggattcttgaCAGTTGTTTCATATGAAGAGATTGTTAAGTCAAGATAATCAATAGGAGAAATCAATATGATCGTTTCTGACATCGAAGCTAACGCCCTCTTAGAGAGCGTCACTAAGTTCCACTGCGGGGt                                                                                                                                                                                     <  1:264581‑M2/121‑1 (MQ=255)`  
                                                                                                                             `gacaggcgttcgtaatctaaagtgccgctttactggtgatactagtatcgctggctacatggaatacaacaaggaaaccggatggcttgaaccatcaagttactcaggggaagaagagtcacactcagagtcaacagactggtccaacgacactgacttctgacaggattcttgaCAGTTGTTTCATATGAAGAGATTGTTAAGTCACGATAATCAATAGGAGAAATCAATATGATCGTTTCTGACATCGAAGCTAACGCCCTCTTAGAGAGCGTCACTAAGTTCCACTGCGGGGTTATCt                                                                                                                                                                                <  1:89974‑M2/126‑1 (MQ=255)`  
                                                                                                                                `gcctaaccttgtcctcgttcgtattctcaagtgccgctttactggtgatactggtatcgctggctacatggaatacaacaaggaaaccggatggcttgaaccatcaagttactcaggggaagaagagtcacactcagagtcaacagactggtccaacgacactgacttttgaCAGTTGTTTCATATGAAGAGATTGTTAAGTCACGATAATCAATAGGAGAAATCAATATGATCGTTTCTGACATCGAAGCTAACGCCCTCTTAGAGAGCGTCACTAAGTTCCACTGCGGGGTTATCTACg                                                                                                                                                                             >  1:77672‑M2/173‑301 (MQ=255)`  
                                                                                                                                  `ggtgggctcggagatgtgtataagagacaggtgatactggtatcgctggctacatggaatacaacaaggaaaccggatggcttgaaccatcaagttactcaggggaagaagagtcacactcagagtcaacagactgttccaacgacactgacttctgacaggattcttgaCAGTTGTTTCATATGAAGAGATTGTTAAGTCACGATAATCAATAGGAGAAATCAATATGATCGTTTCTGACATCGAAGCTAACGCCATCTTAGAGAGCGTCACTAAGTTCCACTGCGGGGTTATCTACGac                                                                                                                                                                           <  1:192450‑M2/131‑1 (MQ=255)`  
                                                                                                                                  `ggctcggagatgtgtataagagacagactggtgatactggtatcgctggctacatggaatacaacaaggaaaccggatggcttgaaccatcaagttactcaggggaagaagagtcacactcagagtcaacagactggtccaacgacactgacttctgacaggattcttgaCAGTTGTTTCATATGAAGAGATTGTTAAGTCACGATAATCAATAGGAGAAATCAATATGATCGTTTCTGACATCGAAGCTAACGCCCTCTTAGAGAGCGTCACTAAGTTCCACTGCGGGGTTATCTACGac                                                                                                                                                                           <  1:252367‑M2/131‑1 (MQ=255)`  
                                                                                                                                   `cgttcgtatgctcaattgacgcttgactggggagactggaatcgctggctaaatgtaatacaacaaggaaaccggatggcttgaaccatcaagttactcagtggaagaagagtcacactcagagtcaacagactggtccaactacactgacttctgacaggatgcttgaCAGTTGTTTCATATGAAGAGATTGTTAAGTCACGATAATCAATAGGAGAAATCAATATGATCGTTTCTGACATCGAAGCCAACGCCCTCTTAGAGAGCGTCACTAAGTTCCACTGCGGGGTTATCTACGact                                                                                                                                                                          <  1:199940‑M2/132‑1 (MQ=255)`  
                                                                                                                                        `ttattctcaagtgccgctttactggtgatactggtatcgctggctacatggaatacaacaaggaaaccggatggcttgaaccatcaagttactcaggggaagaagagtcacactcagagtcaacagactggtccaacgacactgacttctgacaggattcttgaCAGTTGTTTCATATGAAGAGATTGTTAAGTCACGATAATCAATAGGAGAAATCAATATGATCGTTTCTGACATCGAAGCTAACGCCCTCTTAGAGAGCGTCACTAAGTTCCACTGCGGGGTTATCTACGACTACTcc                                                                                                                                                                     <  1:187306‑M2/137‑1 (MQ=255)`  
                                                                                                                                        `gtatgctcaagtgccgctttactggagatactggtatcgctggctacatggaatacaacaaggaaaccggatggcttgaaccatcaagttactcatgggaagaagagtcacactcagagtcaacagactggtccaacgacactgacttctgacaggattcttgaCAGTTGTTTCATAAGAAGAGATTGTTAAGTCACGATAATCAATAGGAGAAATCAATATGATCGTTTCTGACATCGAAGCTAACGCCCTCTTAGAGAGCGTCACTAAGTTCCACTGCGGGGTTATCTACGACTACTcc                                                                                                                                                                     <  1:42102‑M2/137‑1 (MQ=255)`  
                                                                                                                                          `attatcaagtgccgctttactggtgatactggtatcgctggctacatggaatacaacaaggaaaccggatggcttgaaccatcaagttactcaggggaagaagagtcacactcagagtcaacagactggtccaacgacactgacttctgacaggattcttgaCAGTTGTTTCATATGAAGAGATTGTTAAGTCACGATAATCAATAGGAGAAATCAATATGATCGTTTCTGACATCGAAGCTAACGCCCTCTTAGAGAGCGTCACTAAGTTCCACTGCGGGGTTATCTACGACTACTCCAc                                                                                                                                                                   <  1:40130‑M2/139‑1 (MQ=255)`  
                                                                                                                                             `taagagacaggctttactggtgatactggtatcgctggctacatggaatacaacaaggaaaccggatggcttgaaccatcaagttactcaggggaagaagagtcacactcagagtcaacagactggtccaacgacactgacttctgacaggattcttgaCAGTTGTTTCATATGAAGAGATTGTTAAGTCACGATAATCAATAGGAGAAATCAATATGATCGTTTCTGACATCGAAGCTAACGCCCTCTTATAGAGCGTCACTAAGTTCCACTGCGGGGTTATCTACGACTACTCCACCGc                                                                                                                                                                <  1:280027‑M2/142‑1 (MQ=255)`  
                                                                                                                                             `ctcaagtgccgctttactggtgatactggtatcgctggctacatggaatacaacaaggaaaccggatggcttgaaccatcaagttactcaggggaagaagagtcacactcagagtcaacagactggtccaacgacactgacttctgacaggtttcatgaCAGTTGTTTCATATGAAGAGATTGTTAAGTCACGATAATCAATAGGAGAAATCAATATGATCGTTTCTGACATCGAAGCCAACGCCCTCTTAGAGAGCGTCACTAAGTTCCACTGCGGGGTTATCTACTACTACTCCACCGc                                                                                                                                                                >  1:300791‑M2/160‑301 (MQ=255)`  
                                                                                                                                                          `ttattggtgatactggtatcgctggctacatggaatacaacaaggaaaccggatggcttgaaccatcaagttactcaggggaagaagagtcacactcagagtcaacagactggtccaacgacactgacttctgacatgattcttgaCAGTTGTTTCATATGAAGAGATTGTTAAGTCACGATAATCAATAGGAGAAATCAATATGATCGTTTCTGACATCGAAGCTAACGCCCTCTTAGATAGCGTCACTAAGTTCCACTGCGGGGTTATCTACGACTACTCCACCGCTGAGTACGTAAGc                                                                                                                                                   <  1:282115‑M2/155‑1 (MQ=255)`  
                                                                                                                                                               `ggtgatactggtatcgctggctacatggaatacaacaaggaaaccggatggcttgaaccatcaagttactcaggggaagaagagtcacactcagagtcaacagactggtccaacgacactgacttctgacaggattcttgaCAGTTGTTTCATATGAAGAGATTGTTAAGTCACGATAATCAATAGGAGAAATCAATATGATCGTTTCTGACATCGAAGCTAACGCCCTCTTAGAGAGCGTCACTAAGTTCCACTGCGGGGTTATCTACGACTACTCCACCGCTGAGTACGTAAGCTACCg                                                                                                                                              >  1:16049‑M2/142‑301 (MQ=255)`  
                                                                                                                                                                    `tactggtatcgctggctacatggaatacaacaaggaaaccggatggcttgaaccatcaagttactcaggggaagaagagtcacactcagagtcaacagactggtccaacgacactgacttctgacaggattcttgaCAGTTGTTTCATATGAAGAGATTGTTAAGTCACGATAATCAATATGAGAAATCAATATGATCGTTTCTGACATCGAAGCCAACGCCCTCTTAGAGAGCGTCACTAAGTTCCACTGCGGggttatctacgactactccaccgcctgtctcttatacacatctccga                                                                                                                                         >  1:173648‑M2/137‑278 (MQ=255)`  
                                                                                                                                                                       `tggtagcgatggctaaatggaatacaaaaaggaaaccggatggcttgaaccatcaagttactcaggagaagaagagtcacactcagagtcaacagactggtccaacgacactgacttctgacaggtttcttgaCAGTTGTTTCATATGAAGAGATTGTTAAGTCACGATAATCAATAGGAGAAATCAATATGATCGTTTCTGACATCGAAGCTAACGCCCTCTTAGAGAGCGTCACTAAGTTCCACTGCGGGGTTATCTACGACTACTCCACCGCTGAGTACGTAAGCTACAGTCCGAGTg                                                                                                                                      <  1:132115‑M2/168‑1 (MQ=255)`  
                                                                                                                                                                       `gtgtataagagacagcatggaatacaacaaggaaaccggatggcttgaaccatcaagttactcaggggaagaagagtcacactcagagtcaacagactggtccaacgacactgacttctgacaggattcttgaCAGTTGTTTCATATGAAGAGATTGTTAAGTCACGATAATCAATAGGAGAAATCAATATGATCGTTTCTGACATCGAAGCCAACGCCCTCTTAGAGAGCGTCACTAAGTTCCACTGCGTGGTTATCTACGACTACTCCACCGCTGAGTACGTAAGCTACCGTCCAAGTg                                                                                                                                      <  1:152508‑M2/168‑1 (MQ=255)`  
                                                                                                                                                                        `ggtatcgctggctacatggaatacaacaaggaaaccggatggcttgaaccatcaagttactcaggggaagaagagtcacactcagagtcaacagactggtccaacgacactgacttctgacaggattcttgaCAGTTGTTTCATATGAAGAGATTGTTAAGTCACGATAATCAATAGGAGAAATCAATATGATCGTTTCTGACATCGAAGCTAACGCCCTCTTAGAGAGCGTCACTAAGTTCCACTGCGTGGTTATCTACGACTACTCCACCGCTGAGTACGTAAGCTACCGTCCGAGTGa                                                                                                                                     >  1:276206‑M2/133‑301 (MQ=255)`  
                                                                                                                                                                                      `catggaatacaacaaggaaaccggatggattgaaccatcaagttactcaggggaagaagagtcacactcagagtcaacagactggtccaacgacactgacttctgacaggattcttgaCAGTTGTTTCATATGAAGAGATTGTTAAGTCACGATAATCAATAGGAGAAATCAATATGATCGTTTCTGACATCGAAGCTAACGCCCTCTTAGAGAGCGTCACTAAGTTCCACTGCGGGGTTATCTACGACTACTCCACCGCTGAGTACGTAAGCTACCGTCCGATTGACTTCGGTGCGTATc                                                                                                                       >  1:177264‑M2/119‑301 (MQ=255)`  
                                                                                                                                                                                          `gaatacaacaaggaaaccggatggcttgaaccatcaagttactcaggggaagaagagtcacactcagagtcaacagactggtccaacgacactgacttctgacaggtttcttgaCAGTTGTTTCATATGAAGAGATTGTTAAGTCACGATAATCAATAGGAGAAATCAATATGATCGTTTCTGACATCGAAGCTAACGCCCTCTTAGAGAGCGTCACTAAGTTCCACTGCGGGGTTATCTACGACTACTCCACCGCTGAGTACGTAAGCTACCGTCCGAGTGACTTCGGTGCGTATCtggt                                                                                                                   >  1:239003‑M2/115‑300 (MQ=255)`  
                                                                                                                                                                                                    `aggaaaccggatggcttgaaccatcaagttactcaggggaagaagagtcacactcagagtcaacagactggtccaacgacactgacttctgacaggattcttgaCAGTTGTTTCATATGAAGAGATTGTTAAGTCACGATAATCAATAGGAGAAATCAATATGATCGTTTCTGACATCGAAGCTAACGCCCTCTTAGAGAGCGTCACTAAGTTCCACTGCGGGGTTATCTACGACTACTCCACCGCTGAGTACGTAAGCTACCGTCCGAGTGACTTCGGTGCGTATCTGGATGCGCTGGaa                                                                                                         <  1:179693‑M2/197‑1 (MQ=255)`  
                                                                                                                                                                                                        `aaccggatggcatgaaccatcaaggtactcaggggaagaagagtcacacgtagagtcaacagactggtccaacgacactgacttctgacaggtttcttgaCAGTGGTTTCATATGAAGAGATTGTTAAGGCACGATAATCAATAGGAGAAATCAAGATGATCGTTTCTGACATCGAAGCTAACGCCCTCTTAGAGAGCGTCACTAAGTTCCACTGCGGGGTTATCTACGACTACTCTACCGCTGAGTACGTAAGCTACCGTCCGAGTGACTTCGGTGCGTATCTGGATGCGCTGGAAGCCg                                                                                                     <  1:318251‑M2/201‑1 (MQ=255)`  
                                                                                                                                                                                                         `accggatggcttgaaccatcaagttactcaggggaagaagagtcacactcagagtcaacagactggtccaacgacactgacttctgacaggattcttgaCAGTTGTTTCATATGAAGAGATTGTTAAGTCACGATAATCAATAGGAGAAATCAATATGAGCGTTTCTGACATCGAAGCTAACGCCCTCTTAGAGAGCGTCACTAAGTTCCACTGCGGGGTTATCTACGACTACTCCACCGCTGAGTACGTAAGCTACCGTCCGAGTGACTTCGGTGCGTATCTGGATGCGCTGGAAGCCGa                                                                                                    <  1:241423‑M2/202‑1 (MQ=255)`  
                                                                                                                                                                                                            `ggatggcttgaaccatcaagttactcaggggaagaagagtcacactcagagtcaacagactggtccaacgacactgacttctgacaggattcttgaCAGTTGTTTCATATGAAGAGATTGTTAAGTCACGATAATCAATAGGAGAAATCAATATGATCGTTTCTGACATCGAAGCTAACGCCCTCTTAGAGAGCGTCACTAAGTTCCACTGCGGGGTTATCTACGACTACTCCACCGCTGAGTACGTAAGCTACCGTCCGAGTGACTTCGGTGCGTATCTGGATGCGCTGGAAGCCGAGGt                                                                                                 <  1:215333‑M2/205‑1 (MQ=255)`  
                                                                                                                                                                                                                   `ttgaaccatcaagttactcaggggaagaagagtcacactcagagtcaacagactggtccaacgacaatgacttctgacaggattcttgaCAGTTGTTTCATATGAAGAGATTGTTAAGTCACGATAATCAATAGGAGAAATCAATATGATCGTTTCTGACATCGAAGCTAACGCCCTCTTAGAGAGCGTCACTAAGTTCCACTGCGGGGTTATCTACGACTACTCCACAGCTGAGTACGTAAGCTACCGTCCGAGTGACTTCGGTGCGTATCTGGATGCGCTGGAAGCCGAGGTTGCACGa                                                                                          <  1:296114‑M2/212‑1 (MQ=255)`  
                                                                                                                                                                                                                            `agacagactcaggtgaagaagagtcacactcagagtcaacagactggtccaacgacactgacttctgacaggattcttgaCAGTTGTTTCATATGAAGAGATTGTTAAGTCACGATAATCAATAGGAGAAATCAATATGATCGTTTCTGACATCGAAGCTAACGCCCTCTTAGAGAGCGTCACTAAGTTCCACTGCGGTGTTATCTACGACTACTCCACCGCTGAGTACGTAAGCTACCGTCTGAGTGACTTCGGTGCGTATCTGGATGCGCTGGAAGCCGAGGTTGCACGAGGCGGTCtt                                                                                 <  1:121733‑M2/221‑1 (MQ=255)`  
                                                                                                                                                                                                                                `ttactcaggggaagaagagtcacactcagagtcaacagactggtccaacgacactgacttctgacaggattcttgaCAGTTGTTTCATATGAAGAGATTGTTAAGTCACGATAATCAATAGGAGAAATCAATATGATCGTTTCTGACATCGAAGCTAACGCCCTCTTAGAGAGCGTCACTAAGTTCCACTGCGGGGTTATCTACGACTACTCCACCGCTGAGTACGTAAGCTACCGTCCGAGTGACTTCGGTGCGTATCTGGATGCGCTGGAAGCCGAGGTTGCACGAGGCGGTCTTATtg                                                                             <  1:166212‑M2/225‑1 (MQ=255)`  
                                                                                                                                                                                                                                  `actcaggggaagaagagtcacactcagagtcaacagactggtccaacgacactgacttctgacaggattcttgaCAGTTGTTTCATATGAAGAGATTGTTAAGTCACGATAATCAATAGGAGAAATCAATATGATCGTTTCTGACATCGAAGCTAACGCCCTCTTAGAGAGCGTCACTAAGTTCCACTGCGGGGTTATCTACGACTACTCCACCGCTGAGTACGTAAGCTACCGTCCGAGTGACTTCGGTGCGTATCTGGATGCGCTGGAAGCCGAGGTTGCACGAGGCGGTCTTATtgtg                                                                           >  1:324316‑M2/75‑301 (MQ=255)`  
                                                                                                                                                                                                                                  `actcaggggaagaagagtcacactcagagtcaacagactggtccaacgacactgacttctgacaggattcttgaCAGTTGTTTCATATGAAGAGATTGTTAAGTCACGATAATCAATAGGAGAAATCAATATGATCGTTTCTGACATCGAAGCTAACGCCCTCTTAGAGAGCGTCACTAAGTTCCACTGCGGGGTTATCTACGACTACTCCACCGCTGAGTACGTAAGCTACCGTCCGAGTGACTTCGGTGCGTATCTGGATGCGCTGGAAGCCGAGGTTGCACGAGGCGGTCTTATtgtg                                                                           >  1:222981‑M2/75‑301 (MQ=255)`  
                                                                                                                                                                                                                                   `ctcaggggaagaagagtcacactcagagtcaacagactggtccaacgacactgacttctgacaggattcttgaCAGTTGCTTCATATGAAGAGATTGTTAAGTCACGATAATCAATAGGAAACATCAATATGATCGTTTCTGACATCGAAGCTAACGCCCTCTTAGAGAGCGTCACTAAGTTCCACTGCGGGGTTATCTACGACTACTCCACCGCTGAGTACGTAAGCTACCGTCCGAGTGACTTCGGTGCGTATCTGGATGCGCTGGAAGCCGAGGTTGCACGAGGCGGTCTTATTgtgt                                                                          <  1:313652‑M2/228‑1 (MQ=255)`  
                                                                                                                                                                                                                                     `gtataagagacaggtcacactcagagtcaacagactggtccaacgacactgacttctgacaggattcttgaCAGTTGTTTCATATGAAGAGATTGTTAAGTCACGATAATCAATAGGAGAAATCAATATGATCGTTTCTGACATCGAAGCTAACGCCCTCTTAGAGAGCGTCACTAAGTTCCACTGCGGGGTTATCTACGACTACTCCACCGCTGAGTACGTAAGCTACCGTCCGAGTGACTTCGGTGCGTATCTGGATGCGCTGGAAGCCGAGGTTGCACGAGGCGGTCTTATTGTGTTc                                                                        <  1:230418‑M2/230‑1 (MQ=255)`  
                                                                                                                                                                                                                                       `ggggaagaagagtcacactcagagtcaacagactggtccaacgacactgacttctgacaggtttcttgaCAGTTGTTTCATATGAAGAGATTGTTAAGTCACGATAATCAATAGGAGAAATCAATATGATCGTTTCTGACATCGAAGCTAACGCCCTCTTAGAGAGCGTCACTAAGTTCCACTGCGGGGTTATCTACGACTACTCCACCGCTGAGTACGTAAGCTACCGTCCGAGTGACTTCGGTGCGTATCTGGATGCGCTGGAAGCCGATGTTGCACGAGGCGGTCTTATTGTGTGCca                                                                      >  1:288773‑M2/70‑301 (MQ=255)`  
                                                                                                                                                                                                                                          `gaagaagagtcacactcagagtcaacagactggtccaacgacactgacttctgacaggattcttgaCAGTTGTTTCATATGAAGAGATTGTTAAGTCACGATAATCAATAGGAGAAATCAATATGATCGTTTCTGACATCGAAGCTAACGCCCTCTTAGAGAGTGTCACTAAGTTCCACTGCGGGGTTATCTACGACTACTGCACCGCTGAGTACGTAAGCTACCGTCCGAGTGACTTCGGTGCGTATCTGGATGCGCTGGAAGCCGAGGTTGCACGAGGCGGTCTTATTGTGTTCCACaa                                                                   <  1:140680‑M2/235‑1 (MQ=255)`  
                                                                                                                                                                                                                                          `gaagaagagtcacactcagagtcaacagactggtccaacgacactgacttctgacaggattcttgaCAGTTGTTTCATATGAAGAGATTGTTAAGTCACGATAATCAATAGGAGAAATCAATATGATCGTTTCTGACATCGAAGCTAACGCCCTCTTAGAGAGCGTCACTAAGTTCCACTGCGGGGTTATCTACGACTACTCCACCGCTGAGTACGTAAGCTACCGTCCGAGTGACTTCGGTGCGTATCTGGATGTGCTGGAAGCCGAGGTTGCACGAGGCGGTCTTATTGTGTTCCACaa                                                                   >  1:115015‑M2/67‑301 (MQ=255)`  
                                                                                                                                                                                                                                          `gaagaagagtcacactcagagtcaacagactggtccaacgacactgacttctgacaggattcttgaCAGTTGTTTCATATGAAGAGATTGTTAAGTCACGATAATCAATAGGAGAAATCAATATGATCGTTTCTGACATCGAAGCTAACGCCCTCTTAGAGAGCGTCACTAAGTTCCACTGCGGGGTTATCTACGACTACTCCACCGCTGAGTACGTAAGCTACCGTCCGAGTGACTTCGGTGCGTATCTGGATGCGCTGGAAGCCGAGGTTGCACGAGGCGGTCTTATTGTGTTCCACaa                                                                   <  1:306070‑M2/235‑1 (MQ=255)`  
                                                                                                                                                                                                                                          `aatgtgtataagagacaggagtcaacaggctggtccaacgacactgacttctgacaggattcttgaCAGTTGTTTCATATGAAGAGATTGTTAAGTCACGATAATCAATAGGAGAAATCAATATGATCGTTTCTGACATCGAAGCTAACGCCCTCTTAGAGAGCGTCACTAAGTTCCACTGCGGGGTTATCTACGACTACTCCACCGCTGAGTACGTAAGCTACCGTCCGAGTGACTTCGGTGCGTATCTGGATGCGCTGGAAGCCGAGGTTGCACGAGGCGGTCTTATTGTGTTCCACaa                                                                   <  1:126522‑M2/235‑1 (MQ=255)`  
                                                                                                                                                                                                                                                `gagtcacactcagcgtcaacagactggtccaacgacactgacttctgacaggattcttgaCAGTTGTTTCATATGAAGAGCTTGTTAAGTCACGATAATCAATAGGAGAAATCAATATGATCGTTTCTGACATCGAAGCTAACGCCCTCTTAGAGAGCGTCACTAAGTTCCACTGCGGGGTTATCTACGACTACTCCACCGCTGAGTACGTAAGCTACCGTCCGAGTGACTTCTGTGCGTATCTGTATGCGCTGGAAGCCGAGGTTGCACGAGGCGGTCttatggtgtgccctttctcttt                                                             >  1:273544‑M2/61‑291 (MQ=255)`  
                                                                                                                                                                                                                                                `gagtcacactcagagtccacagactggtccaacgacactgacttcttcccgtcttcttgaCAGTTGTTTCATATGAAGAGATTGTTACGTCACGATACTCAATAGGAGAAATCAATAGGATCGTTTCTGACATCGAGGCTAACACCCTCTTATAGAGCTTCACTAAGTTTCACTGCGGGGTTGTCTACGACTACTCCACCGCTGCGTACGTAAGCTACCGTCCGAGTGACTTCGGGACGTATCTGGAGGCGCTAGAAGCCGAGGTTGCACGAGGCTGGCTTCTTTTGTTCCACGACTGTca                                                             >  1:154483‑M2/61‑301 (MQ=255)`  
                                                                                                                                                                                                                                                `gagtcacactcagagtcaacagactggtccaacgacactgacttctgacaggattcttgaCAGTTGTTTCATATGAAGAGATTGTTAAGTCACGATAATCAATAGGAGAAATCAATATGATCGTTTCTGACATCGAAGCTAACGCCCTCTTAGAGAGCGTCACTAAGTTCCACTGCGGGGTTATCTACGACTACTCCACCGCTGAGTACGTAAGCTACCGTCCGAGTGACTTCGGTGCGTATCTGGATGCGCTGGAAGCCGAGGTTGCACGAGGCGGTCTTATTGTGTTCcacaacctgtc                                                             >  1:218060‑M2/61‑296 (MQ=255)`  
                                                                                                                                                                                                                                                  `gtcatactcagagtcaacagactggtccaacgacactgacttctgacaggattcttgaCAGTTGTTTCATATGAAGAGATTGTTAAGTCACGATAATCAATAGGAGAAATCAATATGATCGTTTCTGACATCGAAGCTAACGCCCTCTTAGAGAGCGTCACTAAGTTCCACTGCGGGGTTATCTACGACTACTCCACCGCTGAGTACGTAAGCTACCGTCCGAGTGACTTCGGTGCGTATCTGGATGCGCTGGAAGCCGAGGTTGCACGAGGCGGTCTTATTGTGTTCCACAACGGTcaca                                                           >  1:156619‑M2/59‑301 (MQ=255)`  
                                                                                                                                                                                                                                                  `gtcacactcagagtcaacagactggtccaacgacactgacttctgacaggattcttgaCAGTTGTTTCATATGAAGAGATTGTTAAGTCACGATAATCAATAGGAGAAATCAATATGATCGTTTCTGACATCGAAGCTAACGCCCTCTTAGAGAGCGTCACTAAGTTCCACTGCGGGGTTATCTACGACTACTCCACCGCTGAGTACGTAAGCTACCGTCCGAGTGCCTTCGGTGCGTATCTGGATGCGCTGGAAGCCGAGGTTGCACGAGGCGGTATTATTGTGTTCCACAACGGtcacc                                                           >  1:171094‑M2/59‑300 (MQ=255)`  
                                                                                                                                                                                                                                                      `cactcagagtcaacagactggtccaacgacactgacttctgacaggattcttgaCAGTTGTTTCATATGAATAGATTGTTAAGTCACGATAATCAATAGGAGAAATCAATATGATCGTTTCTGACATCGAAGCTAACGCCCTCTTAGAGAGCGTCACTAAGTTCCACTGCGGGGTTATCTACGACTACTCCACCGCTGAGTACGTAAGCTACCGTCCGAGTGACTTCGGTGCGTATCTGGATGCGCTGGAAGCCGAGGTTGCACGAGGCGGTCTTATTGTGTTCCACAACGGTCACAAGTa                                                       <  1:62866‑M2/247‑1 (MQ=255)`  
                                                                                                                                                                                                                                                      `cactcagagtcaacagactggtccaacgacactgacttctgacaggattcttgaCAGTTGTTTCATATGAAGAGATTGTTAAGTCACGATAATCAATAGGAGAAATCAATATGATCGTTTATGACATCGAAGCTAACGCCCTCTTAGAGAGCGTCACTAAGTTCCACTGCGGGGTTATCTACGACTACTCCACCGCTGAGTACGTAAGCTACCGTCCGAGTGACTTCGGTGCGTATCTGGATGCGCTGGAAGACGAGGTTGCACGAGGCGgtcttattgtgttccacctgtctcttataca                                                       >  1:342179‑M2/55‑287 (MQ=255)`  
                                                                                                                                                                                                                                                            `gtataagagacagggtccaacgacactgacttctgacaggattcttgaCAGTTGTTTCATATGAAGAGATTGTTAAGTCACGATAATCAATAGGAGAAATCAATATGATCGTTTCTGACATCGAAGCTAACGCCCTCTTAGAGAGCGTCACTAAGTTCCACTGCGGGGTTATCTACGACTACTCCACCGCTGAGTACGTAAGCTACCGTCCGAGTGACTTCTGTGCGTATCTGGATGCGCTGGAAGCCGAGGTTGCACGAGGCGGTCTTATTGTGTTCCACAACGGTCACAAGTATGACGt                                                 <  1:182889‑M2/253‑1 (MQ=255)`  
                                                                                                                                                                                                                                                            `gagtcaacagactggtccaacgacactgacttctgacaggattcttgaCAGTTGTTTCATATGAAGAGATTGTTAAGTCACGATAATCAATAGGAGAAATCAATATGATCGTTTCTGACATCGAAGCTAACGCCCTCTTAGAGAGCGTCACTAAGTTCCACTGCGGGGTTATCTACGACTACTCCACCGCTGAGTACGTAAGCTACCGTCCGAGTGACTTCGGTGCGTATCTGGATGCGCTGGAAGCCGAGGTTGCACGAGGCGGTCTTATTGTGTTacacaacggtcacacgctgtctct                                                 >  1:254999‑M2/49‑291 (MQ=255)`  
                                                                                                                                                                                                                                                            `gagtcaacagactggtccaacgacactgacttctgacaggattcttgaCAGTTGTTTCATATGAAGAGATTGTTAAGTCACGATAATCAATAGGAGAAATCAATATGATCGTTTCTGACATCGAAGCTAACGCCCTCTTAGAGAGCGTCACTAAGTTCCACTGCGGGGTTATCTACGACTACTCCACCGCTGAGTACGTAAGCTACCGTCCGAGTGACTTCGGTGCGTATCTGGATGCGCTGGAAGCCGAGGTTGCACGAGGCGGTCTTATTGTGTTCCACAACGGTCACAAGTATGACGt                                                 <  1:205147‑M2/253‑1 (MQ=255)`  
                                                                                                                                                                                                                                                              `gtcaacagactggtccaacgacactgacttctgacaggattcttgaCAGTTGTTTCATATGAAGAGATTGTTAAGTCACGATAATCAATAGGAGAAATCAATATGATCGTTTCTGACATCGAAGCTAACGCCCTCTTAGAGAGCGTCACTAAGTTCCACTGCGGGGTTATCTACGACTACTCCACCGCTGAGTACGTAAGCTACCGTCCGAGTGACTTCGGTGCGTATCTGGATGCGCTGGAAGCCGAGGTTGCACGAGGCGGTCTTATTGTGTTCCACAACGGTCACAAGTATGACGTTc                                               >  1:20823‑M2/47‑301 (MQ=255)`  
                                                                                                                                                                                                                                                                `caacagactggtccaacgacactgacttctgacaggattcttgaCAGTTGTTTCATATGAAGAGATTGTTAAGTCACGATAATCAATAGGAGAAATCAATATGATCGTTTCTGACATCGAAGCTAACGCCCTCTTAGAGAGCGTCACTAAGTTCCACTGCGGGGTTATCTACGACTACTCCACCGCTGAGTACGTAAGCTACCGTCCGAGTAACTTCGGTGCGTATCTGGATGCGCTGGAAGCCGAGGTTGCACGAGGCGGTCTTATTGTGTTCCACAACGGTCACAAGACTGACGTTCCt                                             >  1:44185‑M2/45‑301 (MQ=255)`  
                                                                                                                                                                                                                                                                    `agactggtccaacgacactgacttctgacaggtttcttgaCAGTTGTTGCATATGAAGAGATTGTTAAGTCACGATAATCAATAGGAGAAATCAATATGATCGTTTCTGACATCGAAGCTAACGCCCTCTTAGAGAGCGTCACTAAGTTCCACTGCAGGGTTATCTACGACTACTCCACCGCTGAGTACGTAAGCTACCGTCCGAGTGACTTCGGTGCGTATCTGGATGCGCTGGAAGCCGAGGTTGCACGAGGCGGTCTTATTGTGTTCCACAACGGTCACAAGTATGACGTTCCTGCAt                                         <  1:212226‑M2/261‑1 (MQ=255)`  
                                                                                                                                                                                                                                                                     `gactggtccaacgacactgacttctgacaggattcttgaCAGTTGTTTCATATGAAGAGATTGTTAAGTCACGATAATCAATAGGAGAAATCAATATGATCGTTTCTGACATCGAAGCTAACGCCCTCTTAGAGAGCGTCACTAAGTTCCACTGCGGGGTTATCTACGACTACTCCACCGCTGAGTACGTAAGCTACCGTCCGAGTGACTTCGGTGCGTATCTGGATGCGCTGGAAGCCGAGGTTGCACGAGGCGGTATTATTGTGTTCCACAACGGTCACAAGTATGACGTTCCTGCAtt                                        >  1:258477‑M2/40‑301 (MQ=255)`  
                                                                                                                                                                                                                                                                              `ttatcagagactgtctgacaggcttcttgaCAGTTGTTTCATATGAAGAGATTGTTAAGTCACGATAATCAATAGGAGAAATCAATATGATCGTTTCTGACATCGAAGCTAACGCCCTCTTAGAGAGCGTCACTAAGTTCCACTGCGGGGTTATCTACGACTACTCCACCGCTGAGTACGTAAGCTACCGTCCGAGTGACTTCGGTGCGTATCTGGATGCGCTGGAAGCCGAGGTTGCACGAGGCGGTCTTATTGTGTTCCACAACGGTCACAAGTATGACGTTCCTGCATTGACCAAACt                               <  1:109545‑M2/271‑1 (MQ=255)`  
                                                                                                                                                                                                                                                                                `cgacactgacttctgacaggattcttgaCAGTTGTTTCATATGAAGAGATTGTTAAGTCACGATAATCAATAGGAGAAATCAATATGATCGTTTCTGACATCGAAGCTAACGCCCTCTTAGAGAGCGTCACTAAGTTCCACTGCGGGGTTATCTACGACTACTCCACCGCTGAGTACGTAAGCTACCGTCAGAGTGACTTCGGTGCGTATCTGGATGCGCTGGAAGCCGAGGTTGCACGAGGCGGTCTTATTGTGTTCCACAACGGTCACAAGTATGACGTTCCTGCATTGACCAAACTgg                             >  1:158454‑M2/29‑301 (MQ=255)`  
                                                                                                                                                                                                                                                                                              `gacaggattcttgaCAGTTGTTTCATATGAAGAGATTGTTAAGTCACGATAATCAATAGGAGAAATCAATATGATCGTTTCTGACATCGAAGCTAACGCCCTCTTAGAGAGCGTCACTAAGTTCCACTGCGGGGTTATCTACGACTACTCCACCGCTGAGTACGTAAGCTACCGTCCGAGTGACTTCGGTGCGTATCTGGATGCGCTGGAAGCCGAGGTTGCACGAGGCGGTCTTATTGTGTTCCACAACGGTCACAAGTATGACGTTCCTGCATTGACCAAACTGGCAAAGTTGCAATTg               <  1:335170‑M2/287‑1 (MQ=255)`  
                                                                                                                                                                                                                                                                                                       `cttgaCAGTTGTTTCATATGAAGAGATTGTTAAGTCACGATAATCAATAGGAGAAATCAATATGATCGTTTCTGACATCGAAGCTATCGCCCTCTTAGAGAGCGTCACTAAGTTCCACTGCGGGGTTATCTACGACTACTCCACCGTTGAGTACGTAAGCTACCGTCCGAGTGACTTCGGTGCGTATCTGGATGCGCTGGAAGCCGAGGTTGCACGAGGCGGTCTTATTGTGTTCCACAACGGTCACAAGTATGACGTTCCTGCATTGACCAAACTGGCAAAGTTGCAATTGAACCGagag      >  1:340314‑M2/6‑301 (MQ=255)`  

                                                                                                                                                                                                                                                                                                            `|`                                                                                                                                                                                                                                                                                                             
`ATGAAGCTAAGACTCGCTCCGCTGTTCACATCTTGAAGAACTTAGGCTGGACTTGGACAAGACAGACTGGCTGGAAGAAACCAGAAGTTACCAAGCTGAGTCATAAGGTGTTCGATAAGGACACTATGACCCACATCAAGGCTGGTGATTGGGTTAAGGTTGACATGGGAGTTGTTGGTGGATACGGCTACGTCCGCTCAGTTAGTGGCAAATATGCACAAGTGTCATACATCACAGGTGTTACTCCACGCGGTGCAATCGTTGCCGATAAGACCAACATGATTCACACAGGTTTCTTGACAGTTGTTTCATATGAAGAGATTGTTAAGTCACGATAATCAATAGGAGAAATCAATATGATCGTTTCTGACATCGAAGCTAACGCCCTCTTAGAGAGCGTCACTAAGTTCCACTGCGGGGTTATCTACGACTACTCCACCGCTGAGTACGTAAGCTACCGTCCGAGTGACTTCGGTGCGTATCTGGATGCGCTGGAAGCCGAGGTTGCACGAGGCGGTCTTATTGTGTTCCACAACGGTCACAAGTATGACGTTCCTGCATTGACCAAACTGGCAAAGTTGCAATTGAACCGAGAGTTCC  >  NC_001604.1/13997‑14596`

  

Alignment Legend

Aligned base mismatch/match (shaded by quality score): `ATCG/ATCG` `< 3 ≤` `ATCG/ATCG` `< 11 ≤` `ATCG/ATCG` `< 25 ≤` `ATCG/ATCG` `< 36 ≤` `ATCG/ATCG` `< 38 ≤` `ATCG/ATCG`

Unaligned base: `atcg`    Masked matching base: `atcg`    Alignment gap: `‑`    Deleted base: `‑`
